# First epidemiological survey of *Leishmania infantum* in the domestic ferret (*Mustela putorius furo*) in a canine leishmaniosis endemic area using serology and PCR

**DOI:** 10.1186/s13071-022-05517-y

**Published:** 2022-10-17

**Authors:** María Magdalena Alcover, Jacobo Giner, Judit Rabasedas, Xavier Roca-Geronés, Maite Verde, Antonio Fernández, Cristina Riera, Roser Fisa, Sergio Villanueva-Saz

**Affiliations:** 1 Departament de Biologia, Facultat de Farmacia, Salut i Medi Ambient, Universitat de Barcelona, Barcelona, Spain; 2 Laboratorio de Inmunopatología Clínica, Facultad de Veterinaria, Universidad de Zaragoza, Zaragoza, Spain; 3 Clínica Veterinaria Menescalia, Actor Ismael Merlo, 5, Valencia, Spain; 4 Departamento de Patología Animal, Facultad de Veterinaria, Universidad de Zaragoza, Zaragoza, Spain; 5 Instituto Agroalimentario de Aragón-IA2 (Universidad de Zaragoza-CITA), Zaragoza, Spain

**Keywords:** ELISA, Ferret, *Leishmania infantum*, Prevalence, PCR, Serology, Western blot

## Abstract

**Background:**

Leishmaniosis, a vector-borne disease caused by *Leishmania infantum*, is one of the most important parasitic zoonoses in Europe. The transmission cycle of leishmaniosis is maintained by both domestic and wild animals. However, few data are available on the role of wild mammals in transmitting the parasite in the European Mediterranean basin. As feline leishmaniosis, diagnosis of the infection in ferrets can be a challenge, the use of different serological and molecular methods combined is a recommended approach. Our aim was to investigate the prevalence of infection of *L. infantum* in apparently healthy domestic ferrets (*Mustela putorius furo*) in an endemic region of Spain (Community of Valencia), using serological and molecular methods and to evaluate the results comparing the different techniques.

**Methods:**

The prevalence of *Leishmania* infection was studied in domestic ferrets. Blood was collected from each animal for serology and molecular analysis. Two serological methods, enzyme-linked immunosorbent assay (ELISA) and western blot (WB), were used for the detection of *L. infantum* antibodies, and real-time polymerase chain reaction (qPCR) was used for the detection of *L. infantum* DNA.

**Results:**

Blood samples from 102 apparently healthy ferrets were analyzed. In the serological study, 25.5% of the animals tested positive by western blot, and 9.0% by enzyme-linked immunosorbent assays. The seroprevalence of *L. infantum* infection, based on a positive result in any serological test, was 28.4% (95% confidence interval [CI] 20.6–S37.9%). No kinetoplast DNA (kDNA) was detected by qPCR in peripheral blood samples from the ferrets tested.

**Conclusions:**

The immunological response revealed by these tests indicates that the ferrets are exposed to repeated inoculations with the endemic parasite *L. infantum*. Although the low population of domestic ferrets means their reservoir potential is limited in the absence of a primary host, it would be of interest to carry out further studies using xenodiagnosis to determine whether they are accidental or reservoir host species capable of spreading infection.

**Graphical Abstract:**

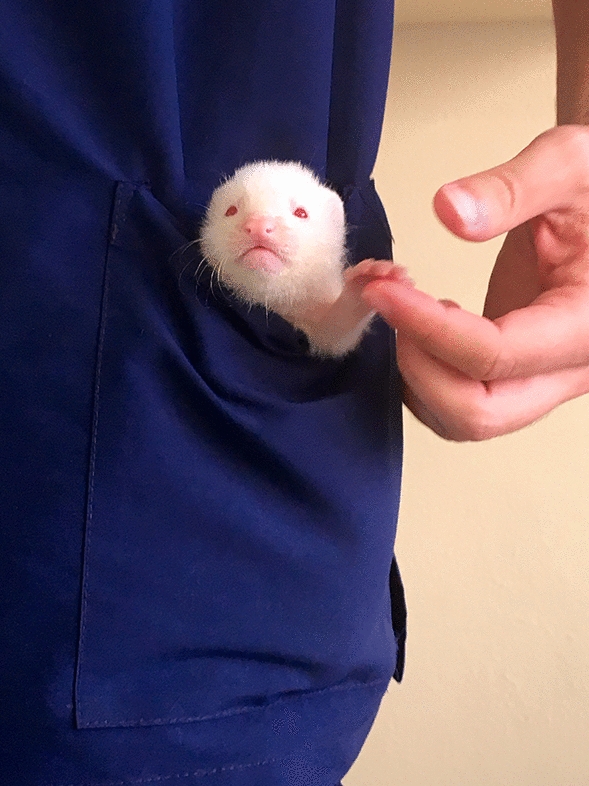

**Supplementary Information:**

The online version contains supplementary material available at 10.1186/s13071-022-05517-y.

## Background

Leishmaniosis, a vector-borne disease caused by *Leishmania infantum*, is one of the most important parasitic zoonoses in Europe [1]. In the past decade, several wild animals, including lagomorphs and canids, have been described as playing a significant role in the transmission cycle of *L. infantum*. Moreover, an increasing number of reports suggest that leishmaniosis is not restricted to dogs (main reservoir) but may also affect other mammalian and avian species [2]. For instance, immunological positivity to *L. infantum* in cats from European endemic areas suggests they are frequently exposed to the parasite [3–6] and subjected to repeated inoculations. Additionally, several studies have detected *L. infantum* kinetoplast DNA (kDNA) in species of the Mustelidae family [7–10] and anti-*L. infantum* antibodies in American [11] and European mink in Spain [12].

The most common clinical signs of leishmaniosis described in ferrets* (Mustela putorius furo*) are peripheral lymphadenomegaly and cutaneous lesions, including papular and ulcerative dermatitis [13]. Frequently observed clinicopathological abnormalities are hyperproteinemia with hyperglobulinemia and polyclonal gammopathy [13]. In ferrets, clinical data for leishmaniosis are still very scarce. Moreover, there is no epidemiological information about the prevalence and seroprevalence of leishmaniosis in domestic ferrets in areas endemic for *L. infantum*, including the Community of Valencia (Spain), where seropositive clinically sick animals have been reported [14, 15].

Epidemiological surveys are an important step in establishing the presence/absence of a pathogen in a specific region, and the results are of great value to clinicians when infections are potentially underdiagnosed, as in the case of leishmaniosis in domestic ferrets. Epidemiological studies of canine and feline leishmaniosis have combined serological and molecular methods for maximum diagnostic efficiency, an approach that could also be applied to ferrets [3, 16–19]. However, serological methods need to be adapted and validated for each species to avoid negative results in seropositive animals [13].

Given the absence of studies on leishmaniosis in ferrets in the Community of Valencia, where cases have been recently reported, the present study aimed (1) to provide the first epidemiological data on the role of domestic ferrets in the epidemiology of *L. infantum* infection in an endemic region; (2) to investigate the prevalence of *L. infantum* infection in domestic ferrets using serology and quantitative molecular assays (quantitative polymerase chain reaction [qPCR]); and (3) to evaluate the screening results of apparently healthy domestic ferrets living in an endemic region by comparing serological and qPCR data.

## Methods

### Study areas, ferrets, and sampling

Blood samples were collected from September 2019 to February 2021 from 102 ferrets in different towns of the Province of Valencia (39° 99 28′12.864″ N, 0° 22′36.48″ W), an area on the east coast of the Iberian Peninsula with a high incidence of canine leishmaniosis (50–100/1000 dogs/year) [20, 21]. Based on epidemiological studies, 17.1% of dogs are seropositive in the Community of Valencia in Spain [21, 22]. However, the presence of human cutaneous leishmaniosis outbreak has also been detected in the Valencia region [22]

Before sampling, information was obtained about each animal regarding age, cohabitation with a dog, lifestyle (indoor, outdoor, or mixed) and gender, and a complete physical examination was carried out to establish health status (sick versus healthy). Whenever possible, one ml of blood was collected aseptically by cranial cava venipuncture from each ferret. The collected volume was divided equally between a sterile blood collection tube (to obtain the serum) and a second tube containing ethylenediaminetetraacetic acid (EDTA) anticoagulant (for molecular analysis). EDTA-blood and separated sera were stored at −20 °C until processing. Routine laboratory tests, such as a complete blood count and biochemistry profile, were not performed.

In total, 102 client-owned ferrets were sampled. A single sample was obtained from 94 ferrets. The other eight client-owned ferrets were tested an additional one (*n* = 5) to two (*n* = 3) different times during the study period. A total of 113 serum samples were included.

### Diagnostic serological tests

Detection of specific antibodies was performed using two in-house serological techniques: enzyme-linked immunosorbent assay (ELISA) and western blot (WB).

#### Detection of *L. infantum* antibodies by a quantitative ELISA

The ELISA was performed on all sera as described previously, with some modifications [14]. Briefly, each plate was coated with 20 µg/ml of crude antigen obtained from *L. infantum* promastigote forms (MHOM/MON-1/LEM 75) in 0.1 M carbonate/bicarbonate buffer (pH 9.6) and incubated overnight at 4 °C. Next, 100 µl of cat sera, diluted 1:200 in phosphate-buffered saline (PBS) containing 0.05% Tween 20 (PBST) and 1% dry skimmed milk (PBST-M), was added to each well. The plates were incubated for 1 h at 37 °C in a moist chamber. Then they were washed, and 100 µl of protein A conjugated to horseradish peroxidase (Thermo Fisher Scientific, Waltham, MA, USA) diluted 1:8000 in PBST-M was added. The plates were incubated for 1 h at 37 °C in the moist chamber and were washed again with PBST and PBS as described above. The substrate solution (ortho-phenylenediamine) and stable peroxide substrate buffer (Thermo Fisher Scientific, Waltham, MA, USA) was added per well and developed for 20 ± 5 min at room temperature in the dark. The reaction was stopped by adding 2.5 M H_2_SO_4_ to each well. Absorbance values were read at 492 nm in an automatic microELISA reader (Multiskan ELISA reader, Labsystems, Midland, Canada).

As a positive control (calibrator), each plate included serum from a ferret from Spain diagnosed with leishmaniosis, confirmed by a positive culture, and as a negative control, serum from a healthy, non-infected ferret. The same positive control serum was used for all assays and plates, with a constant inter-assay variation of < 10%. Plates with an inter-assay variation of > 10% were discarded. All samples and controls were run in duplicate. The cut-off was set to 0.180 optical density units (OD) (mean + 3 standard deviations of values from 30 indoor ferrets from northern Spain), and results above this value were considered positive.

#### Detection of *L. infantum* antibodies by WB

Anti-*Leishmania* antibodies were detected by WB using a whole antigen of *L. infantum* promastigotes (MHOM/FR/78/LEM75 zymodeme MON-1), as described by Alcover et al. [3], with some modifications. The protocol used for WB is based on the technique described by Aisa et al. [23], with sensitivity of 95.8% and specificity of 100% in dogs. Moreover, the specificity has been analyzed including a group of healthy cats (n = 20) from a non-endemic area (Switzerland) with a value of 100%. Antigen electrophoresis on 0.1% sodium dodecyl sulfate (SDS)-15% polyacrylamide gels together with molecular mass protein standards (low-range standard; Bio-Rad, Hercules, CA, USA) was performed on a Mini-Gel AE-6400 Dual Mini Slab Kit (Atto, Bunkyo-ku, Japan).

Gels were run at 100 V for 1 h at room temperature. Polypeptides were transblotted onto nitrocellulose sheets (0.45-mm pore size, HAWP 304 FO; Millipore, Bedford, MA, USA), which were blocked with 20 mM Tris, 0.13 mM NaCl, pH 7.6 (TS), and 5% skimmed milk overnight at 4 °C.

The sheets were washed in TS and introduced into a multiscreen apparatus (Mini-PROTEAN II; Bio-Rad, Hercules, CA, USA). Sera were diluted 1:200 in TS-1% skimmed milk and 0.2% Tween 20. Then 500 µl of each sample was introduced into each channel of the multiscreen apparatus and incubated for 2 h at 37 °C. Bound immunoglobulins were developed by incubation with a 1:1000 dilution of protein A peroxidase conjugate (Thermo Fisher Scientific, Waltham, MA, USA) for 1 h. After the sheets were washed three times with TST and a final time with TS, color was developed with 4-chloro-1-naphthol substrate (Thermo Fisher Scientific, Waltham, MA, USA). Based on our experience and the literature, a serum sample was considered positive when immunoreactivity against the *L. infantum* antigen fraction 14 and /or 16 kDa was observed, and indeterminate when molecular weight bands of 18, 20, 24, 28, 30, 36, 38, and 46 kDa appeared, as reported previously [3].

#### Detection of *L. infantum* DNA by qPCR

DNA was extracted from 200 μl of mammalian blood using the High Pure PCR Template Preparation Kit (Roche Applied Science, Mannheim, Germany), which allows genomic DNA to be isolated rapidly and easily from a wide variety of sample materials. All extractions were performed following the manufacturer’s instructions, and multiple PCR templates were obtained in minutes using efficient High Pure spin columns.

The detection and quantification of *Leishmania* kDNA was carried out by amplification of kinetoplast minicircle DNA sequences by qPCR [13]. Each amplification was performed in triplicate in 10 μl of reaction mixture containing 1× iTaq Supermix with ROX (Bio-Rad, Hercules, CA, USA), 15 pmol of direct primer Leim1 (5′-CTT TTC TGG TCC TCC GGG TAG G-3′), 15 pmol of reverse primer Leim2 (5′- CCA CCC GGC CCT ATT TTA CAC CAA-3′), 50 pmol of the labeled TaqMan probe Leim3 (5′- FAM-TTT TCG CAG AAC GCC CCT ACC CGC TAMRA-3′), and 2.5 μl of sample DNA.

The ABI Prism 7900 HT thermocycler (Applied Biosystems, Waltham, MA, USA) was used at 94 °C and 55 °C cycling over 40 cycles with a FAM detector. A non-template control was used in each run as the qPCR negative control. A 10-fold dilution series of DNA from promastigotes (MHOM/ES/04/BCN-61, *L. infantum)* was used for calibration (serial dilution from 10^5^ parasites/ml to 10^−3^ parasites/ml), allowing the plotting of a standard curve. The qPCR was considered positive when the threshold cycle (Ct) was lower than 40 and when the amplification was detected in all the replicates [3, 24, 25].

### Statistical analysis

Data collected for the entire population were analyzed using descriptive statistics. Associations between *L. infantum* and the recorded variables were analyzed. The significance of this difference was assessed using the Chi-square or Fisher’s exact test. A value of *P* ≤ 0.05 was considered significant. The SPSS v.22 software grogram was used (IBM Corporation, Armonk, NY, USA).

## Results

### Animals studied

All the tested ferrets (*n* = 102; 49 females and 53 males) had a mixture of coat colors, and none had been surgically neutered. All ferrets were classified as apparently healthy, with no systemic signs of disease found in the general physical examination. The mean age of the animals was 4 years (ranging from 1 to 8), and they were classified as young (< 2 years), adult (from ≥ 2 years to ≤ 6 years), or senior (> 6 years). None of the ferrets had been treated with a long-acting topical anti-parasitic repellent against sand flies (Table 1).

**Table 1:** Evidence of contact with *L. infantum* using ELISA and western blot

Variable	Animals studied (%)	ELISA		Western blot			Positive animals for both techniques (%)
		Positive (%)	Negative (%)	Positive (%) ^a^	Indeterminate (%) ^b^	Negative (%) ^c^	
Sex							
Female	49 (48.0)	4 (8.2)	45 (91.8)	15 (30.6)	2 (4.1)	32 (65.3)	16 (32.7)
Male	53 (52.0)	5 (9.4)	48 (90.6)	11 (20.8)	7 (13.2)	35 (66.0)	13 (24.5)
Total	102	9 (8.8)	93 (91.2)	26 (25.5)	9 (8.8)	67 (65.7)	29 (28.4)
Age							
Young	15 (14.7)	0 (0)	15 (100)	4 (26.7)	2 (13.3)	9 (60)	4 (26.7)
Adult	48 (47.1)	5 (10.4)	43 (89.6)	16 (33.3)	4 (8.3)	28 (58.3)	17 (35.4)
Senior	39 (38.2)	4 (10.23)	35 (89.7)	6 (15.4)	3 (7.7)	30 (76.9)	8 (20.5)
Housing shelter							
Inside	55 (53.9)	4 (7.2)	51 (92.72)	10 (18.2)	3 (5.5)	42 (76.3)	13 (23.6)
Outside	6 (5.9)	1 (16.7)	5 (83.33)	2 (33.3)	2 (33.3)	2 (33.3)	2 (33.3)
Mixed	41 (40.2)	4 (9.8)	37 (90.24)	14 (34.2)	4 (9.8)	23 (56.1)	14 (34.2)
Cohabitation with a dog							
Yes	28 (27.5)	3 (10.7)	25 (89.3)	8 (28.6)	2 (7.1)	18 (64.3)	8 (28.6)
No	74 (72.6)	6 (8.1)	68 (91.9)	18 (24.3)	7 (9.5)	49 (66.2)	21 (28.4)

^a^ 14 and/or 16 kDa bands were present

^b^ Band patterns observed but not for 14 and/or 16 kDa

^c^ No band observed

No significant association (*P* > 0.05) was detected between *Leishmania* positivity and gender (male/female), age (young, adult, senior), cohabitation with a dog, or lifestyle (indoor, outdoor, or mixed). All statistical analysis can be found in Additional file 1: Table S1.

For the serological study, a total of 113 serum samples were analyzed, 11 of which were obtained from the eight seropositive animals that were followed up. Molecular tests were applied to 62 peripheral blood samples, seven of which belonged to five seropositive animals that were followed up. EDTA-blood could not be obtained from some of the domestic ferrets (*n* = 47) due to their small size and the absence of an anesthetic procedure during blood extraction.

### Serology and qPCR for *L. infantum*

**Fig. 1:**
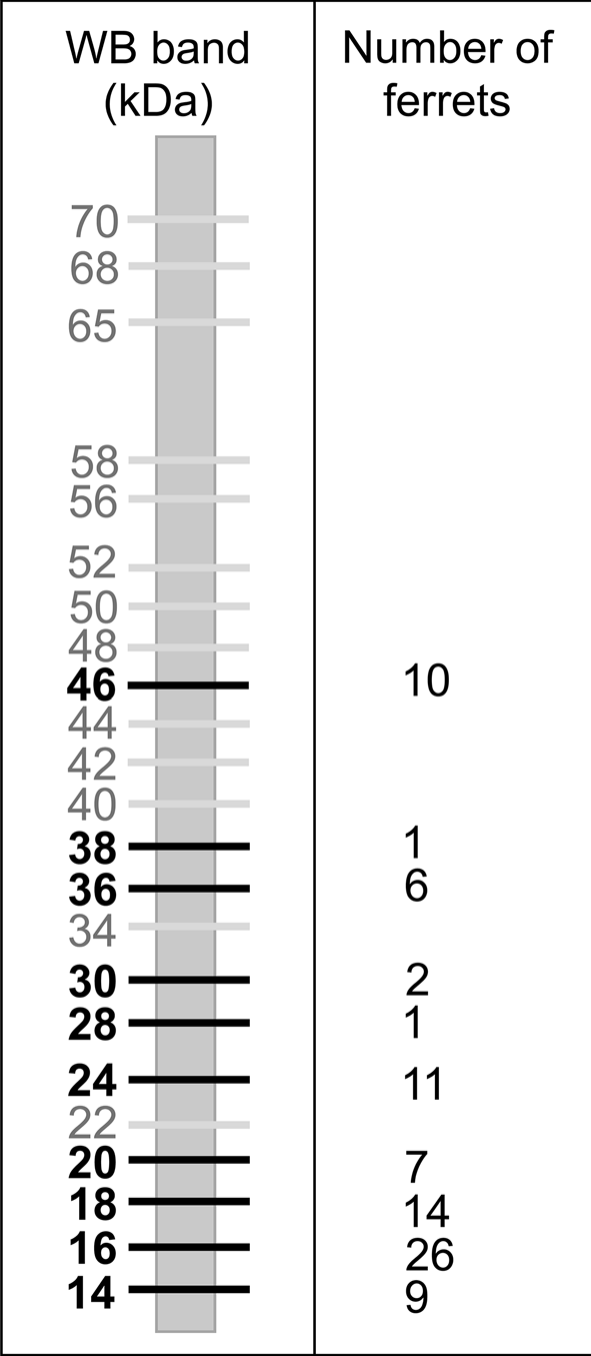
Western blot results in 102 domestic ferrets from Valencia (Spain). A positive result was considered when bands of 14 and/or 16 kDa were observed. When bands of 18, 20, 24, 28, 30, 36, 38, and 46 kDa appeared, the results were regarded as undetermined

In the serological study, 100% of the animals were analyzed by both ELISA and WB. By ELISA, nine positive animals were detected, the seropositive rate being 8.8% (95% CI 4.5–16.1%). Using WB, 26 were positive, which constitutes a seropositivity of 25.5% (95% CI 18.0–34.8%), and nine ferrets (8.8%) gave indeterminate results (i.e., no bands were observed at 14 and/or 16 kDa). The WB data for sensitivity to the *L. infantum* antigen are provided in Fig. 1. Among the 26 positive ferrets, bands were observed at both 14 and 16 kDa for nine animals, at 16 kDa and other molecular weights for 12, and only at 16 kDa for five ferrets.

Of the nine animals diagnosed as indeterminate by WB, eight were negative by other techniques, and one with a band at 46 kDa tested positive by ELISA. Twenty-nine ferrets (28.4%) tested positive by at least one of the three techniques (95% CI 20.6–37.9%). Twenty of these were diagnosed as positive only by WB, and three only by ELISA (including one of the three WB indeterminate sera), whereas six ferrets tested positive by both serological techniques. Eight of the 29 positive animals were followed up, four of them testing negative in the second sample; two of the four were analyzed only by serological techniques and two by ELISA, WB, and PCR. The WB result for one of these four animals was indeterminate, yielding a band only at 20 kDa.

During the follow-up of eight animals originally found positive by WB, three remained positive by WB in the second analysis, and only one (M5706) tested positive in a third analysis (Table 2). One of the three ferrets (M5931) was also positive by ELISA, and again tested positive by both serological techniques in the second analysis (ELISA titer of 0.241 OD and WB band at 16 kDa); however, in the subsequent follow-up, both tests were negative (Table 2). The third of the three ferrets (M6188) tested negative in the second analysis. This animal was classified as apparently healthy ferret without evidence clinical signs detected during physical examination. In this sense, it is possible to detect seasonal variation in anti-*Leishmania* antibodies in dogs [26] and ferrets [27] in endemic areas of canine leishmaniosis. Finally, all the asymptomatic ferrets with full blood samples, previously screened for *Leishmania* DNA, tested negative.

**Table 2:** Follow-up of ferrets that had contact with *L. infantum*

	First determination			First follow-up			Second follow-up		
Identification	PCR	ELISA (OD)	WB (kDa)	PCR	ELISA (OD)	WB (kDa)	PCR	ELISA (OD)	WB (kDa)
M5798	−	−	+ (14/16/18/24/36/46)	NP	−	−	NP	NP	NP
M6314	−	−	+ (14/16/18)	−	−	−	NP	NP	NP
M5706	−	−	+ (16/20/46)	−	−	+ (16/118/24)	NP	NP	NP
M5741	−	−	+ (16)	−	−	Undetermined (20)	NP	NP	NP
M6626	−	−	+ (16/18)	NP	−	−	NP	NP	NP
M6188	NP	−	+ (16/18)	NP	+ (0.226)	+ (14/16/18/20/24)	−	−	−
M5412	−	−	+ (16)	NP	+ (0.186)	−	NP	NP	NP
M5931	NP	+ (0.201)	+ (14/16)	−	+ (16)	−	−	−	−

*NP* not performed, *OD* optical density; +: positive; −: negative

## Discussion

In this study, 102 domestic ferrets in the Community of Valencia (Spain) were tested for *L. infantum* infection using serological and molecular techniques. The rate of seropositivity was high by both ELISA and WB, although the number of positive cases detected by WB was statistically significantly higher (*P* < 0.008). WB is known to be a more sensitive method than ELISA, which is more specific [28]. Generally used as a confirmatory test of ELISA results, WB is more difficult to perform and requires higher laboratory skills. However, the results of the present study do not reflect these differences in sensitivity and specificity.

The detection of *L. infantum* kDNA by qPCR has been previously evaluated in a range of matrices [3, 24, 25]. In veterinary medicine, it is known, especially in dogs, that blood is not the best matrix for *Leishmania* qPCR [29]. Noninvasive samples including oral and conjunctival swabs, hair, or saliva have been evaluated, but there is a lack of studies that support the usefulness of such samples applied in clinical practice as follow-up and clinical prognostic parameters [30–32]. In Spain, blood samples from stray cats have been tested by qPCR to detect the parasite, and variations in prevalence between studies and endemic regions have been reported [3]. Parasite detection using qPCR assays based on highly repetitive gene loci or extrachromosomal kDNA sequences is restricted to the genus level. In the present study, we used extremely sensitive genus-specific primers to be able to detect asymptomatic animals. Even so, the qPCR results of peripheral blood samples were all negative, possibly because the parasite load in the circulatory system was insufficient for detection by this technique or was nonexistent. *Leishmania* protozoa mainly multiply in macrophages of the skin and spleen, and therefore in mild infections their levels in blood are low [33, 34]. Thus, although peripheral blood samples are easy to obtain and are minimally invasive, they proved unsuitable for detecting kDNA of *Leishmania* spp. in asymptomatic ferrets, which may have low or unstable parasitemia over time. A study analyzing *Leishmania* infection in mustelids using tissue macerates reported high percentages of kDNA detection by qPCR [7], suggesting that this type of sample is more suitable for genetic analysis, although it is also more invasive. In ferrets, we suspect that *L. infantum* would be more prevalent in organ tissues than in blood. This may imply that ferrets are not efficient *Leishmania* reservoirs and cannot sustain the parasite transmission cycle alone. For further clarification of the nature of *Leishmania* infection in ferrets, xenodiagnostic studies would be useful [3, 35–37].

In general, seroprevalence studies on specific pathogens in animals produce variable results depending on factors such as the geographical location, lifestyle, age, or analytical methodology. The data obtained in the present study provide an estimation of *L. infantum* seroprevalence in domestic ferrets in Spain. Although we obtained data demonstrating ferret exposure to *L. infantum* inoculations, in accord with a previous report [27], the mere presence of antibodies or parasite DNA is not sufficient evidence of reservoir host status [38]. To confirm whether an animal is an accidental or reservoir (primary or secondary) host of *L. infantum* requires xenodiagnosis. Also, in areas with a high density of sand fly vectors and biting rates, if a suitable vertebrate reservoir host species for *Leishmania* is absent or rare, the transmission cycle of the parasite is not sustained in the long term. Such a case has been reported in a peri-urban zoological park in southern Spain, where most sand flies feed on large herbivores that do not act as *Leishmania* reservoirs [37].

Although ferrets may not play a crucial role as reservoir hosts, their involvement in the parasite cycle may still have a significant impact. More studies are needed to elucidate the epidemiological role of domestic ferrets in the spread of leishmaniosis in *L. infantum* endemic areas such as the Community of Valencia in Spain.

## Conclusions

Our serological results revealed that domestic ferrets in the Mediterranean basin are exposed to the endemic parasite *L. infantum*. To our knowledge, this study demonstrates for the first time the seroprevalence and prevalence rates of *L. infantum* in domestic ferrets in an endemic region of Spain. Further prevalence surveys in other endemic regions, using other diagnostic methods and more suitable matrices for molecular analysis, would be useful to clarify the role of this popular pet in the epidemiology of *L. infantum* transmission.

## Supplementary Information

**Additional file 1: Table S1.** Statistical analysis of the positivity to IgG antibodies against *L. infantum* in ferrets in Spain, in relation to sex, age, housing shelter, and cohabitation with a dog.

## Data Availability

The datasets supporting the findings of this article are included within the article and its additional files.

## Abbreviations

Ct: Threshold cycle
EDTA: Ethylenediaminetetraacetic acid
ELISA: Enzyme-linked immunosorbent assay
qPCR: Quantitative real-time PCR
PCR: Polymerase chain reaction
WB: Western blot
