# Supplementary material for: First epidemiological survey of Leishmania infantum in the domestic ferret (Mustela putorius furo) in a canine leishmaniosis endemic area using serology and PCR
Source: Parasit Vectors. 2022 Oct 17;15:372. doi: 10.1186/s13071-022-05517-y (PMC9575300; doi:10.1186/s13071-022-05517-y)
Supplement: Supplementary file 1 — Additional file 1: Table S1. Statistical analysis of the positivity to IgG antibodies against L. infantum in ferrets in Spain, in relation to sex, age, housing shelter, and cohabitation with a dog. [file 13071_2022_5517_MOESM1_ESM.docx]

**Additional file 1: Table S1.** Statistical analysis of the positivity to IgG antibodies against *Leishmania infantum* in ferrets in Spain, in relation to sex, age, housing shelter, cohabitation with a dog, and serological test.

| Variable | Enzyme-linked immunosorbent assay | Western blot |
| --- | --- | --- |
| Sex | *χ*2 = 0.05, *df* = 1, *P* = 0.82 | *χ*2 = 3.37, *df* = 2, *P* = 0.19 |
| Age | *χ*2 = 1.73, *df* = 2, *P* = 0.43 | *χ*2 = 4.06 *df* = 4, *P* = 0.40 |
| Housing shelter | *χ*2 = 0.67, *df* = 2, *P* = 0.72 | *χ*2 = 9.45, *df* = 4, *P* = 0.05 |
| Cohabitation with a dog | *χ*2 = 0.17, *df* = 1, *P* = 0.70 | *χ*2 = 0.26, *df* = 2, *P* = 0.88 |
